# Notes and News

**Published:** 1901-09-21

**Authors:** 


### NOTES AND NEWS.

The Prince of Wales's Hospital Fund has received £100 from Lord Grimthorpe, completing an annual subscription of £200, and £100 from the Alliance Assurance Company.

Smallpox is still clinging tenaciously to the Metropolis, and its spread is rather more rapid. Upwards of 136 cases have been notified since the outbreak. It has made its appearance in a locality it has favoured before, that of Lisson Grove, a very crowded and squalid area.

The British Red Cross Committee have made a grant of £1,000 to the Soldiers' and Sailors' Help Society. It is to be utilised for the benefit of invalids from South Africa. The society has at the request of the War Office undertaken to provide for soldiers on sick furlough, and the Red Cross Committee have set aside £5,000 to assist in the work.

Examination of statistics relating to the population of Hong Kong reveals the extraordinary high infant mortality amongst the Chinese community. It seems hardly possible to believe that out of every 1,000 Chinese born in the colony, only 72 survive the first year of existence. This is what, however, the reports on statistics show. The European infant mortality in Hong Kong is high, but that of the Chinese points to an absolutely criminal neglect at the least. Definite sanitary regulations are gradually improving the condition of Hong Kong, but apparently it needs other steps also if the Chinese population is to be preserved.

It appears to be now decided to make a good many alterations in the House of Commons for the greater comfort of the members. But judging from the outline of the scheme to spend £30,000 which has reached us, a very small amount of the money will go towards improving that atmosphere of which we have heard so many complaints. The objections made to it are serious, and indeed the House has lost several useful members who could not face the ordeal of long hours in such badly ventilated quarters. Here surely would be an opportunity of adopting the very best ventilating apparatus and plant to regulate temperature. For the good of the whole country efforts should be made to secure hygienic improvements before or alongside those which affect smoking, dining, and reading-room accommodation.

It is to be very much doubted whether a day out is ever beneficial to the health of children, but lately several outings which have had really disastrous results have been reported. The last mishap occurred at a fête given by the Tilbury Garrison, which resulted in the illness of thirty children, apparently from the food they had eaten.

There is no doubt that public opinion generally is becoming roused against the objectionable habit of spitting. Paris is the latest convert, and attention there is being drawn vigorously to the dangers of the practice. In London public opinion is beginning to take effect. There is certainly less spitting on the tops of omnibuses since the request has been made that people should not do so. It is true that foot passengers now experience a danger which is so common in America, but after all a step is being taken in the right direction, and expectoration on the roads is better than on the floors of vehicles.

The statistics of the Imperial Institute of Experimental Medicine of St. Petersburg, which deal with inoculation for hydrophobia, are interesting reading. It is curious to find how very large a number of cases which have not actually run any real risk present themselves for inoculation. It is often found that the dogs which have bitten patients are not rabid, and in a large number of other cases the skin of the person bitten has not been broken. Amongst what may be described as genuine cases of hydrophobia contagion, the Institute records a mortality of 1·2 per cent.

A special meeting of the council of the Metropolitan Hospital Sunday Fund was held at the Mansion House on Wednesday, 11th inst. The business consisted of the consideration of a letter which was read from Mr. Henry N. Custance, proffering his resignation of the office of secretary, which he has held for 28 years. He has, however, been unable lately to perform the duties owing to a breakdown in health. Eulogistic references were made by Sir Sydney Waterlow (who as Lord Mayor founded the society at the end of 1872), and others to the admirable manner in which Mr. Custance had discharged the work of his position. The council decided to give Mr. Custance six months' further leave of absence from November 1st next, in the hope that at the expiration of that period he would be able to resume the performance of his duties.

General belief has been prevalent amongst the public that, unpleasant as a high wind usually is, it is at any rate healthy. Surgeon-General Harvey's recent report on enteric fever in the British Army in India rather dissipates this idea, at any rate in connection with cities or places where numbers of people congregate. He points out the undeniable fact that in dust is conveyed much offensive matter, and high wind and dust are companions. It is quite understandable that the inhabitants of the Metropolis run extra risks during the time when high winds are scattering microbes in their faces. Bicyclists and automobilists often suffer very severely from dust in their eyes, and it is not wholly due to the irritation produced by the sandy material which enters them. The mischief is often undoubtedly septic. In eye-cases the direct harm caused by dust is easily traced. In most it is not so. It would be interesting to watch whether a large number of persons do not suffer from what is termed "catching a cold" on the first appearance of a high wind in any city.

